# The Use of Herbs and Spices to Improve Food Liking and Diet Quality

**DOI:** 10.1093/nutrit/nuaf249

**Published:** 2026-05-26

**Authors:** John C Peters, James O Hill

**Affiliations:** Anschutz Health and Wellness Center, University of Colorado, Denver, Anschutz Medical Campus, Aurora, CO 80045, United States; Department of Nutrition Sciences, University of Alabama, Birmingham, Birmingham, AL 35233, United States

**Keywords:** culinary spices, food liking, diet quality, fat reduction, sugar reduction, protein intake

## Abstract

We reviewed 7 studies described in 4 publications examining whether culinary spices can improve the liking of foods reduced in fat, saturated fat, energy, sugar, and protein-rich foods in older adults. All studies were randomized, within-subject, crossover studies comparing foods with or without culinary spices. The addition of spices to reduced-fat foods successfully restored liking to levels comparable with full-fat versions for most items tested, although this strategy was less effective for foods for which fat contributes significantly to texture, such as creamy pasta dishes. For sugar reduction, spices were most successful in foods with complex flavor profiles for which sweetness was not the dominant characteristic. In protein studies with older adults, spice addition enhanced flavor intensity and improved acceptance of both meat- and plant-based high-protein meals, although achieving recommended protein targets was more successful with animal-based dishes. These findings demonstrated that culinary spices can serve as an effective tool for improving the liking of foods reformulated to meet dietary guidelines, particularly for fat and saturated fat reduction and in foods with multifaceted flavor profiles, potentially helping consumers adopt healthier eating patterns.

## INTRODUCTION

We conducted a review of 7 studies described in 4 publications^1–4^ that examined whether adding herbs and spices to healthier versions of popular dishes can increase likability and consumption to match their standard counterparts. This article appears as part of the supplement “The Role of Spices and Herbs on Supporting Healthy Diets and Improving Nutritional Status,” sponsored by the McCormick Science Institute.

This report does not present a comprehensive review of the literature about the use of herbs and spices to influence food flavor and consumption. The studies described focused on foods that were reduced in fat, saturated fat, calories, and sugar, and protein-rich foods for adults older than 60 years. The overarching purpose of this work was to better understand whether herbs and spices can be an effective tool for helping Americans meet dietary guidelines by enhancing the flavor and likability of more healthful food preparations.

Nearly half of adults in the United States do not follow the Dietary Guidelines for Americans (DGA; 2020-2025).^5^ Despite decades of advice to reduce added sugar, salt, and saturated fat intake, Americans still consume excessive amounts of these components.^6^ Overconsumption is linked to increased risk of heart disease, type 2 diabetes, certain cancers, metabolic syndrome, and obesity.^6–11^ Additionally, older adults often do not consume sufficient dietary protein to prevent lean muscle mass loss, which is key to preserving health and mobility.^12^

Although there is a wealth of popular literature, such as cookbooks, magazines, and online content, that discusses how herbs and spices can enhance the flavor and appeal of food, these sources do not provide evidence on whether these ingredients can improve the likability and consumption of healthier versions of popular dishes compared to their standard counterparts.

At the time these prior studies were conducted, there was limited scientific evidence addressing whether herbs and spices could enhance the liking and consumption of foods containing healthier levels of saturated fat, added sugar, and protein among older adults. Therefore, we conducted studies examining the potential utility of herbs and spices in enhancing the liking of foods with improved nutritional composition.^1–4^

In this article we present a review of our published studies on the influence of herbs and spices on liking and consumption of the following: (1) reduced-fat foods, (2) reduced-sugar foods, and (3) protein-rich foods for adults older than 60 years.

**The Influence of Herbs and Spices on the Liking of Reduced-Fat Food**

We conducted 3 studies to examine the effects of adding culinary spices on liking and consumption of reduced–fat and saturated fat food items representative of typical breakfast, lunch, and dinner foods.^2^^,^^3^

We used a within-subject, 3-period, 3-condition crossover design with participants randomized to the condition order for all 3 studies. The conditions were the following: full fat (FF), reduced fat with no added herbs and spices (RF), and reduced fat with added herbs and spices (RFS). The reduced-fat foods and total meals were matched for total fat, saturated fat, and calories. Participants were blinded to the test meal condition.

Adults 18-65 years old were enrolled in the studies. Details of the testing procedure can be found in the original publications.^2^^,^^3^ The subjective liking of each meal item and the overall meal were rated on a 9-point hedonic Likert scale from “dislike extremely” to “like extremely.”

Differences in liking scores among food items were analyzed using a linear mixed-effects model consisting of the randomization sequence, test meal period, and test meal condition as fixed-effect predictors. A random subject effect was used. The least square method was used to estimate differences between the types of food tested, and the 95% CIs for this difference were estimated under the model.

We took a number of practical issues into account in designing these studies: We used widely familiar and available foods, a modest number of common herbs and spices, simple food preparation techniques (eg, removing skin from chicken vs leaving the skin on), using leaner cuts of meat, substituting whole milk (3.25% fat) for half and half (11%-15% fat) in some recipes, substituting half and half for whipping cream (30%-36% fat) in other recipes, and using less butter in certain recipes. The foods used for testing were selected such that the fat and saturated fat content could be varied widely by making simple recipe substitutions that anyone could do at home. This allowed us to produce test foods with substantial reductions in total fat, saturated fat, and calories compared to standard preparations.

The calorie, fat, and saturated fat composition of the test foods used in this study are shown in Table 1. Meal items included French toast and sausage for breakfast; roasted chicken, mixed vegetables, and creamy pasta for lunch; and meatloaf with gravy, mixed vegetables, and creamy pasta for dinner. For the different meal items, calories were reduced by 11%-36%, fat by 40%-71%, and saturated fat by 40%-78% compared to the full-fat preparations.

**Table 1.** Total Dietary Fat, Saturated Fat, and Calories by Meal Item, Test Meal, and Test Condition

Meal item	Test meal condition								
	Full fat			Reduced fat^a^					
	Calories (kcal)	Fat (g)	Saturated fat (g)	Calories		Fat		Saturated fat	
				kcal	% Reduction	g	% Reduction	g	% Reduction
French toast	280	8	4	250	11	4.5	44	2	50
Sausage	130	10	4	100	23	6	40	2	50
Total breakfast meal	410	18	8	350	15	10.5	42	4	50
Roasted chicken	220	10	2.5	180	18	6	40	1.5	40
Mixed vegetables	70	4	2.5	45	36	1.5	63	1	60
Creamy pasta	240	14	9	160	33	4	71	2	78
Total lunch meal	530	28	14	385	27	11.5	59	4.5	68
Meat loaf	240	13	5	190	21	7	46	3	40
Total dinner meal^b^	550	31	16.5	395	28	12.5	60	6	64

a Reduced fat (and calorie) with no added spice and reduced fat (and calorie) plus spice.

b Dinner meal included mixed vegetables and creamy pasta, with the same formulation as the lunch meal.

The breakfast meal was French toast and sausage. The FF, RF, and RFS French toast recipes contained the same amounts of eggs, sugar, and bread and were served with 2 teaspoons of maple syrup. The FF French toast was prepared with half and half, while the RF French toast was prepared with whole milk. The base spice was salt. The RFS version of French toast was the same as the RF but with added cinnamon, nutmeg, cinnamon extract, and vanilla extract. Ground pork was used to make the FF sausage patties, and the RF version was made with half as much pork, with 6% added fat and 1% fat ground turkey. The base spices were salt, pepper, and sage. For the RFS sausage, rosemary, thyme, and ground red pepper were added.

For the lunch meal, FF chicken was served with the skin, while RF chicken was served without the skin. The base spices for all food items were salt and pepper. For the RFS chicken, garlic powder, onion powder, fennel seed, smoked paprika, and parsley granules were added. The FF mixed vegetables were prepared in butter sauce, and the FF creamy pasta was prepared with a white sauce made from whipping cream, half and half, and cheese. The RF vegetables were prepared with less butter, and the RF creamy pasta sauce was made with whole milk instead of cream, while the RFS vegetables had added garlic powder, onion powder, and dill weed, and the pasta had added garlic powder, onion powder, Italian seasoning, chervil, and chives.

For the dinner meal, the FF meatloaf was prepared using 80% lean beef, while the RF meatloaf was made with 95% lean beef. Salt and pepper were the base seasonings. The RFS version had the same ingredients as the RF version but with additional garlic and herb seasoning, basil, and oregano. The mixed vegetables and pasta were prepared in the same way as in the lunch study.^2^

### Results

In total, 146 participants completed the breakfast study (69% female, mean age 36.5 years, mean body mass index [BMI] 24.3 kg/m^2^), 150 participants completed the lunch study (70% female, mean age, 36.6 years; mean BMI, 24.3 kg/m^2^), and 148 completed the dinner study (68% female, mean age, 35.9 years; mean BMI, 24.4 kg/m^2^). Subjective liking scores and meal consumption percentages are shown for breakfast, lunch, and dinner in Tables  2-4.

**Table 2.** Mean Likert Scores for Breakfast Meal Items and Percentage of Meal Eaten by Test Meal Condition

Test meal item	Mean Likert score		
	FF	RF	RFS
Overall meal	6.84^a^	6.50^b^	6.82
French toast	7.01^c^	6.43^d^	6.85
Sausage side dish	6.34	6.34	6.41
Mean percentage of meal eaten	83.7	84.1	83.8

Abbreviations: FF, full fat; RF, reduced fat with no added herbs and spices; RFS, reduced fat with added spices.

a *P* = .0016 FF vs RF.

b *P* = .0030 RF vs RFS.

c *P* < .0001 FF vs RF.

d *P* = .0031 RF vs RFS.

**Table 3.** Mean Likert Scores for Lunch Meal Items and Percentage of Meal Eaten by Test Meal Condition

Test meal item	Mean Likert score		
	FF	RF	RFS
Overall meal	6.94^a^	6.35^b^	6.71
Roasted chicken	6.52^c^	5.85^d^	6.49
Vegetable side dish	6.89^e^	6.43	6.66
Pasta side dish	7.47^a^	6.47	6.42^f^
Mean percentage of meal eaten	87.5	85.1	85.5

Abbreviations: FF, full fat; RF, reduced fat with no added herbs and spices; RFS, reduced fat with added spices.

a *P* < .0001 FF vs RF.

b *P* = .0061 RF vs RFS.

c *P* = .0003 FF vs RF.

d *P* = .0005 RF vs RFS.

e *P* = .0006 FF vs RF.

f *P* < .0001 FF vs RFS.

**Table 4.** Mean Likert Scores for Dinner Meal Items and Percentage of Meal Eaten by Test Meal Condition

Test meal item	Mean Likert score		
	FF	RF	RFS
Overall meal	7.05^a^	6.29^b^	6.98
Meatloaf	6.51^c^	6.15^b^	6.76
Vegetable side dish	6.91^d^	6.42^e^	7.02
Pasta side dish	7.33^a^	5.94^b^	6.61^f^
Mean percentage of meal eaten	85.9	83.7	84.9

Abbreviations: FF, full fat; RF, reduced fat with no added herbs and spices; RFS, reduced fat with added spices.

a *P* < .0001 FF vs RF.

b *P* ≤ .0001 RF vs RFS.

c *P* < .02 FF vs RF.

d *P* = .002 FF vs RF.

e *P* = .0002 RF vs RFS.

f *P* < .0001 FF vs RFS.

Overall, the addition of spices improved liking such that the RFS meals were not significantly different from their FF versions. Fat reduction led to a significant reduction in liking of all items compared to the FF versions, although consumption was not affected. Table 4 (dinner) shows that the RFS meatloaf and vegetables had higher liking ratings than the FF versions, although the differences were not significant.

In the lunch meal (Table 3), pasta was the only item for which the liking of the RF and RFS versions remained significantly below that of the FF version. In the dinner meal (Table 4), herbs and spices significantly improved the rating for the RFS pasta compared to the RF preparation but did not significantly improve liking compared to the FF version.

### Discussion of Fat Reduction Studies

The addition of culinary spices was generally effective when the original food item had relatively lower fat content. Spice addition improved the liking of RF roasted chicken, meatloaf, vegetables, and French toast to levels not different from those of the FF versions, whereas spice addition to RF sausage had no effect. The effect of fat reduction on food texture and mouthfeel moderated the effect of culinary spices on liking, as shown by the weak effect on creamy pasta, for which fat dramatically changes mouthfeel and carries important flavor compounds.

Recent studies have demonstrated that herbs and spices can serve as effective tools for improving vegetable and legume liking and consumption among adults,^13–18^ consistent with our findings for mixed vegetables. One study reported that reformulating commonly consumed American foods with herbs and spices to reduce saturated fat was less effective for foods that rely heavily on fat for texture and mouthfeel, consistent with our findings.^19^

Our studies demonstrated that it is possible to cut total fat, saturated fat, and calories in breakfast, lunch, and dinner foods and achieve liking equivalent to full-fat versions by adding herbs and spices to enhance flavor, except for creamy pasta, for which liking depends on fat for expected texture, mouthfeel, and flavor.

**Influence on Liking and Consumption of Adding Spices to Reduced-Sugar Foods**

We conducted 2 studies to investigate the effects of adding culinary spices on liking and consumption of sugar-containing food.^4^ To our knowledge, these are the first studies that have directly compared reduced-sugar foods with or without added culinary spices on liking and consumption. Many studies have examined the negative impact of removing sugar on consumer liking,^20^ and other studies have looked at the acceptability of using artificial sweeteners and other sugar substitutes in place of sugar.^21^ In the first study, the test items were tea and oatmeal served as breakfast, and in the second study, we assessed apple crisp as a mid-afternoon snack. Participants evaluated full-sugar (FS), reduced-sugar (RS), and reduced-sugar plus spice (RSS) versions of the 3 items under 2 test scenarios, absolute liking and relative liking. Absolute liking was evaluated by having participants taste the different foods (in random order) on occasions 1 week apart, and relative liking was assessed by having participants taste all 3 versions of the items at a single sitting. Study procedures and testing conditions were the same as those reported for the studies on fat reduction.^2^^,^^3^

Recipes for the FS, RS, and RSS versions of tea, oatmeal, and apple crisp were the same for both the absolute and relative liking studies. The test foods were selected to create 3 different added sugar conditions: (1) An intrinsic sugar-free item (tea) to which sugar is often added, (2) a food item with minimal intrinsic sugar (oatmeal with dried apples) to which sugar is often added, and (3) an item (apple crisp) with high levels of both intrinsic sugar (from apples) and added recipe sugar.

The total calories and sugar content for the test foods are shown in Table 5. The percentage reduction in sugar for tea was 100%, for oatmeal 35%, and for apple crisp 37%. The tea recipes were made using black tea, with FS containing 2.2 g/serving sugar, while the RS and RSS had no added sugar. Ground ginger, cardamom pods, and whole cloves were added to the RSS tea preparation. Oatmeal recipes consisted of quick oats, dried apples, and salt. The FS recipe contained 14 g/serving of sugar, while the RS and RSS preparations had 7 g/serving. Saigon cinnamon, Jamaican allspice, and vanilla extract were added to the RSS oatmeal recipe. For the mid-afternoon snack, apple crisp filling recipes contained apples and arrowroot. The FS recipe had 7.35 g sugar/serving, while the RS and RSS recipes had 2.9 g/serving. The RSS apple crisp filling also contained Saigon cinnamon. Apple crisp topping recipes contained flour, old-fashioned oats, salt, and butter. The FS apple crisp contained 7.34 g brown sugar and 6.7 g white sugar, while the RS and RSS preparations had half as much of each, 3.67 and 3.35 g of light brown sugar and white sugar, respectively. The RSS apple crisp topping also contained twice the amount of Saigon cinnamon as the FS and RS recipes.

**Table 5.** Total Dietary Sugar and Calories by Test Meal Condition

Meal item	FS	RS, RSS	Reduction from full sugar
Tea			
Total calories (kcal)	10	0	100%
Total sugar (g)	2	0	100%
Oatmeal			
Total calories (kcal)	200	170	15%
Total sugar (g)	17	11	35%
Apple crisp			
Total calories (kcal)	270	230	15%
Total sugar (g)	30	19	37%

Abbreviations: FS, full sugar; RS, reduced sugar; RSS, reduced sugar plus spice.

### Results

Tables  6-8 show liking results for sugar-containing foods with and without culinary spices. For absolute liking (*n* = 160 participants), RS and RSS tea and oatmeal had significantly reduced liking and consumption compared to FS versions. Liking of RSS vs FS apple crisp was not different. For relative liking (*n* = 150 participants), RSS scored significantly better than RS for oatmeal and was not different from FS for apple crisp.

**Table 6.** Absolute Liking Study: Mean Likert Scores for Test Meal Items and Percentage of Meal Eaten by Test Meal Condition

Test meal item	Mean Likert score (% consumed)		
	FS	RS	RSS
Tea	6.27^a^ (40.5%^f^)	5.82 (38.5%)	5.70^b^ (36%)
Oatmeal	7.15^c^ (82%^g^)	6.50 (77%)	6.70^d^ (77.2%^h^)
Apple crisp	7.85^e^ (82.5%)	7.56 (83.1%)	7.66 (84.4%)

Abbreviations: FS, full sugar; RS, reduced sugar; RSS, reduced sugar plus spice.

a *P* = .0079 FS vs RS.

b *P* = .0008 FS vs RSS.

c *P* < .0001 FS vs RS.

d *P* = .0030 FS vs RSS.

e *P* = .0117 FS vs RS.

f *P* = .0095, FS vs RSS.

g *P* = .0033 FS vs RS.

h *P* = .0050 FS vs RSS.

**Table 7.** Relative Liking Study: Mean Likert Scores for Test Meal Items and Percentage of Meal Eaten by Test Meal Condition

Test meal item	Mean Likert score (% consumed)		
	FS	RS	RSS
Tea	6.00^a^ (57.1%^g^)	5.62 (53%)	5.85 (53.7%^h^)
Oatmeal	6.84^b^ (39.7%^i^)	5.69^c^ (31.9%)	6.15^d^ (31.9%^j^)
Apple crisp	7.30^e^ (45.9%)	6.83 (48%)	7.22^f^ (47.8%)

Abbreviations: FS, full sugar; RS, reduced sugar; RSS, reduced sugar plus spice.

a *P* = .03 FS vs RS.

b *P* < .0001 FS vs RS.

c *P* = .0028 RS vs RSS.

d *P* < .0001 FS vs RSS.

e *P* = .0003 FS vs RS.

f *P* = .0023 RS vs RSS.

g *P* = .013 FS vs RS.

h *P* = .04 FS vs RSS.

i *P* < .0001 FS vs RS.

j *P* < .0001 FS vs RSS.

**Table 8.** Percentage of Participants Ranking a Given Test Item as their Best-Liked Version^a^

	Tea (%)	Oatmeal (%)	Apple Crisp (%)
Full sugar (FS)	44	60	36
Reduced sugar (RS)	23	8	22
Reduced sugar spice (RSS)	33	32	42

a Data from Study 2 in which relative ranking was assessed (compare ranking distributions vertically for each column).

### Discussion of Sugar-Reduction Studies

These studies highlight the difficulty in reducing sugar without sweet taste replacement. Most herbs and spices are not strongly sweet and therefore do not contribute sweetness to the flavor profile. Tea and oatmeal are relatively plain foods considerably enhanced by sugar addition. Apple crisp has a more complex ingredient mixture with several components besides sweetness contributing to flavor. Adding ingredients such as cinnamon, allspice, and vanilla to reduced-sugar recipes may preserve a flavorful experience with adequate sweetness.

Culinary herbs and spices may have the greatest potential to help reduce sugar in foods with complex flavor profiles for which sweetness is one of many contributing ingredients. Even small reductions in sugar intake due to spice use, combined with the ability to use herbs and spices to lower fat and salt content, may be beneficial in promoting healthier eating habits.

Reports of recent work include findings that reducing sugar in popular high-sugar foods reduced their liking, and adding herbs and spices could partially or fully restore liking for foods having complex flavor profiles beyond just sweetness,^19^ consistent with our findings.

**Effects of Culinary Spices on Liking and Consumption of Protein-Rich Foods in Community-Dwelling Older Adults**

The current Recommended Dietary Allowance for protein intake is 0.8 g/kg/d for adults older than 18 years.^5^ Studies have shown that protein synthesis is not as efficient in older individuals, and some experts recommend protein intakes of at least 1-1.2 g/kg/d for older adults.^22–25^ We conducted 2 studies^1^ in community-dwelling adults 60 years old and older to examine whether the addition of culinary spices could enhance food liking and protein intake compared to a meal without added spices. Both food liking and flavor intensity were examined as a function of herb and spice addition, given that liking and flavor often correlate with intake. Both a meat-based protein meal (M) and a plant-based protein meal (V) were tested, and recipes were selected to minimize food texture issues (eg, fibrous or tough consistencies) that might affect the ability of older individuals to chew and swallow. Flavor intensity and self-reported declines in smell and taste acuity were assessed to see if enhancing the flavor of high-protein dishes is associated with greater consumption in both individuals with and those without reported sensory loss.

The studies were randomized, 2-period, within-subject crossover in design, whereby different groups of older adults were fed meals 1 week apart containing M or V meals, with or without added culinary spices. The M and V test meals consisted of either chicken salad or bean salad, respectively, and 3 side items: 2 butter lettuce leaves, 2 whole-wheat crackers, and half a cup of seedless grapes. The no-spice-added (NS) preparation of chicken salad consisted of commercially available canned, shredded skinless chicken breast, 2% fat yogurt, mayonnaise, celery, lemon juice, and pepper. The spice-added (S) version of this entrée had the same composition as the NS preparation but with added garlic powder, onion powder, ground mustard seed, and dill. Canned, skinless chicken breast was chosen as the protein source because this food item is a widely available, high-quality, lean protein source that is familiar and liked by most people.

The NS version of the V entrée consisted of garbanzo beans (chickpeas) and brown rice, paired with unflavored soy protein powder, 2% fat yogurt, mayonnaise, celery, lemon juice, water, salt, and pepper. The S version mirrored the NS version but incorporated added culinary spices, including pepper, garlic powder, onion powder, ground mustard seed, parsley, oregano, dill, rosemary, and cumin. Using rice as a complementary ingredient to the beans was crucial for ensuring that the composite protein content of the entrée offered a complete profile of indispensable amino acids. Furthermore, powdered soy protein was added at a level that maintained product acceptance and mouthfeel, thereby enhancing the protein density of the dish.

Participants were served entrée portions based on their body weight to facilitate consumption of at least 0.36 g/kg body weight/meal protein, representing 30% of a daily intake target of 1.2 g/kg body weight, assuming that lunch accounted for approximately 30% of daily energy intake. We targeted delivery of between 18 and 40 g of protein in the meal entrée, depending on body weight. We provided participants with an additional 20% of the entrée target amount to ensure that intake was not constrained to the initial serving size, thus allowing for consumption sufficient to meet the 0.4 g/kg body weight/meal level suggested for supporting optimal protein synthesis in older adults. If individuals consumed the entire entrée amount served, they were free to ask for more.

### Results

Fifty participants completed each study. Total energy consumption and protein intake for the 2 test groups are shown in Table 9. Participants fed both versions of the meat entrée achieved at least 0.40 g/kg body weight of total protein intake per meal (the target for maximizing protein synthesis). Participants fed vegetarian entrées reached only 0.25-0.26 g/kg body weight, primarily due to lower protein density (0.062 g protein/g entrée vs 0.167 g protein/g for meat entrée).

**Table 9.** Energy and Protein Consumption for the M and V Meals

Outcome	Type of entrée	**LS Mean consumption (SEM), no spice**	LS Mean consumption (SEM), spice	*P* for within-entrée comparison, NS vs S	*P* for entrée by recipe interaction
Entrée eaten (Kcal)	Chicken salad	278.15 (11.56)	284.22 (11.56)	.510	.733
	Bean/rice salad	369.27 (27.37)	367.09 (27.37)	.923	
Protein consumed from entrée (g)	Chicken salad	30.24 (1.26)	30.90 (1.26)	.510	.661
	Bean/rice salad	18.24 (1.36)	18.25 (1.36)	.995	
Protein consumed from total meal (g)	Chicken salad	31.97 (1.29)	32.66 (1.29)	.489	.655
	Bean/rice salad	19.97 (1.38)	19.99 (1.38)	.982	
Total protein consumed (g/kg body weight)	Chicken salad	0.40 (0.01)	0.41 (0.01)	.526	.997
	Bean/rice salad	0.25 (0.02)	0.26 (0.02)	.585	

Liking and flavor intensity scores for the M and V entrées are shown in Table 10. Flavor intensity was significantly increased with spice addition for both meal types, and liking improved for the V entrée. This result suggests that spices may be effective for enhancing food flavor and enjoyment in older individuals, potentially encouraging greater food and protein intake.

**Table 10.** Liking and Flavor Intensity Scores for the M and V Entrées

Outcome	Type of entrée	**LS Mean Likert** **score (SEM), no spice**	LS Mean Likert score (SEM), spice	*P* for within-entrée comparison, NS vs S	*P* for entrée by recipe interaction
Liking rating of entrée (after finishing lunch)	Chicken Salad	7.16 (0.20)	7.36 (0.20)	.407	.004
	Bean/rice Salad	4.78 (0.29)	6.04 (0.29)	<.001	
Flavor intensity rating of entrée (after finishing lunch)	Chicken Salad	2.26 (0.13)	2.56 (0.13)	.047	.017
	Bean/rice Salad	1.54 (0.14)	2.36 (0.14)	<.001	

### Discussion of Protein Studies

In these studies, we were able to achieve protein intakes of 30 g/meal and 0.4 g/kg body weight/meal (the targets for maximizing protein synthesis among older adults) among at least two-thirds of participants when the protein source is a high-quality meat protein like chicken. We did not achieve these targets when the protein source was rice and beans, with only 1 in 5 participants meeting the 0.4 g/kg body weight/meal goal for maximizing protein synthesis. One major reason for this result is that the protein density of the V entrée was only 0.062 g protein/g V entrée, whereas the M entrée had a protein density of 0.167 g protein/g M entrée which was over 2.5 times as protein dense. Participants in the V group ate 25% more calories than those in the M group, although this amount was still not able to make up for the difference in protein density. Both groups ate to the same level of satiety. Because most sources of vegetable protein do not have a complete amino acid profile, it is necessary to pair them with complementary protein sources, which can act to dilute the protein density. Since we wanted to feed protein dishes that were easy to chew and swallow, we chose chicken salad and rice and bean salad for the test, which had many other ingredients besides the protein sources. These food form choices further diluted the protein density of the entrées, having the greatest impact on the V entrée. Although it is possible to create meals with vegetable protein that have a greater protein density, it would be more challenging than creating meat- or fish-based dishes due to the limited number of vegetables that have complete amino acid profiles.

We found that flavor intensity was significantly increased with spice addition for both meal types, and liking was improved for the V entrée. This result is encouraging because greater liking is often associated with greater intake, such that protein intake from vegetable protein–rich dishes may be enhanced with spice addition, although we did not observe that outcome in this single-meal test. We also observed that spice addition increased flavor intensity for participants whose sensory acuity had diminished, suggesting that spices may be effective for enhancing food flavor and enjoyment in older individuals, helping encourage greater food and protein intake.

Our findings are consistent with those of other investigations aimed at improving protein intake among elderly participants. Appleton and colleagues^26^^,^^27^ reported that adding palatable sauces to protein dishes increased intake, while other investigators found that providing recipes and herb and spice packets to be used in food preparation improved egg (and protein) consumption.^28^

### Overall Conclusions

The 7 studies described in the 4 publications reviewed here provide support for using culinary spices as a tool to improve diet quality and the liking of foods with healthful nutritional profiles. Adding culinary spices to foods reduced in fat and saturated fat is an effective strategy for improving the liking of these foods, though this effect may be limited for foods for which liking depends on fat-related texture or mouthfeel. Adding spices to reduced-sugar foods can improve liking, but utility may be limited to foods for which the flavor profile is not driven mostly by sweetness. For older adults, culinary spices can enhance flavor even for individuals with declining sensory acuity, increasing the appeal of nutritious foods and encouraging increased consumption.
